# Reduced expression of Toll-like receptor 4 inhibits human breast cancer cells proliferation and inflammatory cytokines secretion

**DOI:** 10.1186/1756-9966-29-92

**Published:** 2010-07-10

**Authors:** Huan Yang, Huiqin Zhou, Ping Feng, Xiaoni Zhou, Huiyan Wen, Xiaofang Xie, Haiying Shen, Xueming Zhu

**Affiliations:** 1 Department of Clinical Laboratory, The Second affiliated Hospital of Soochow University, Suzhou, Jiangsu, China

## Abstract

**Background:**

Tumor cell expression of Toll-like receptors (TLRs) can promote inflammation and cell survival in the tumor microenvironment. Toll-like receptor 4 (TLR4) signaling in tumor cells can mediate tumor cell immune escape and tumor progression, and it is regarded as one of the mechanisms for chronic inflammation in tumorigenesis and progression. The expression of TLR4 in human breast cancer cell line MDA-MB-231 and its biological function in the development and progression of breast cancer have not been investigated. We sought to characterize the expression of TLR1-TLR10 in the established human breast cancer cell line MDA-MB-231, and to investigate the biological roles of TLR4 in breast cancer cells growth, survival, and its potential as a target for breast cancer therapy.

**Methods:**

TLRs mRNA and protein expressions were detected in human breast cancer cell line MDA-MB-231 by RT-PCR, real-time PCR and flow cytometry (FCM). RNA interference was used to knockdown the expression of TLR4 in MDA-MB-231. MDA-MB-231 transfected with the vector pGenesil-1 and the vector containing a scrambled siRNA were as controls. Recombinant plasmids named TLR4AsiRNA, TLR4BsiRNA and TLR4CsiRNA specific to TLR4 were transfected into human breast cancer cell line MDA-MB-231 with Lipfectamine™2000 reagent. TLR4 mRNA and protein expressions were investigated by RT-PCR, real-time PCR, FCM and immunofluorescence after silence. MTT analysis was performed to detect cell proliferation and FCM was used to detect the secretion of inflammatory cytokines in supernatant of transfected cells.

**Results:**

The human breast cancer cell line MDA-MB-231 was found to express TLR1-TLR10 at both the mRNA and protein levels. TLR4 was found to be the highest expressed TLR in MDA-MB-231. TLR4AsiRNA, TLR4BsiRNA and TLR4CsiRNA were found to significantly inhibit TLR4 expression in MDA-MB-231 at both mRNA and protein levels as compared to vector control(vector transfected cells). TLR4AsiRNA mediated the strongest effect. Knockdown of TLR4 gene in MDA-MB-231 resulted in a dramatic reduction of breast cancer cell viability. The cytokines which were secreted by the TLR4 silenced cells, such as IL-6 and IL-8, also decreased significantly as compared with vector control. No significant difference was observed in siRNA control (Recombinant plasmid named ScrambledsiRNA transfected cells) compared to vector control.

**Conclusions:**

These studies identified the expression levels of multiple TLRs in human breast cancer cell line MDA-MB-231 and demonstrated that knockdown of TLR4 could actively inhibit proliferation and survival of breast cancer cells. Taken together, our results suggest RNAi-directed targeting of TLR4 may be a beneficial strategy for breast cancer therapy.

## Introduction

Human toll-like receptors (TLRs), firstly identified in mammalian immune cells, are a family of type I transmembrane proteins comprised of an extracellular domain with a leucine-rich repeat region and an intracellular domain homologous to that of the human interleukin (IL)-1 receptor [1]. TLRs have a powerful capacity to innate immune responses [2] through recognition of pathogen-associated molecular patterns (PAMP) expressed by bacteria and viruses, and host-derived PAMPs [3]. Until now, 11 types of mammalian homologues have been identified and characterized [4].

Recently, new evidence has revealed that TLRs exist in many mouse [5] and human tumors [6-9], such as lung cancer, prostate cancer, neuroblastoma and breast cancer [10]. Although the TLR profile varies in different tumor cells, current evidence indicates that the expression of TLRs and signaling cascade are functionally associated with tumor growth, progression, and invasion. For example, TLR2 signaling has been shown to promote lung cancer cell growth and resistant of apoptosis [11]; TLR3 can directly trigger apoptosis in human cancer cells, such as breast cancer cells [12], TLR2 and TLR9 can promote invasiveness and metastasis through metalloproteases and integrins [13,14].

Breast cancer is one of the common tumors occurring in women which is incurable and ultimately claims the life of the patient with complications. Thus, there is a need for new and effective breast cancer therapies. As TLRs are widely expressed on tumor cells and play important roles in the initiation and progression of cancer, they may thus serve an important target and have an effective perspective on breast cancer treatment.

Therefore, in this study, we aimed to determine which TLRs were expressed in human breast cancer cell line MDA-MB-231 and whether TLR4 played a functional role in the growth and progression of MDA-MB-231. A plasmid vector pGenesil-1 was developed to express a panel of siRNAs directed against TLR4. We planned to exploit the fact that small-interfering RNA (siRNA) can specifically inhibit gene expression with high efficiency [15] and use it as an experimental tool to dissect the cellular pathways that lead to uncontrolled cell proliferation of breast cancer.

## Materials and methods

### Cell line and cell culture

Human breast cancer cell line MDA-MB-231 was purchased from the cell bank of Academia Sinica (Taipei, Taiwan). MDA-MB-231 was grown without antibiotics in 5% CO_2 _at 37°C in RPMI-1640 (Gibco, CA, USA) containing 10% FBS.

### Qualitative RT-PCR

Total RNA was extracted using TRIzol reagent (Invitrogen, CA, USA) and the first-strand cDNA was synthesized according to the manufacturer's instructions using 4 μg total RNA with an oligo-dT primer and the myeloblastosis virus (MLV) reverse transcriptase (Promega, WI, USA). The PCR primers for TLRs (from TLR1 to TLR10) and GAPDH were intron-spanning, and are listed in Table 1. PCR products were analyzed on 1-2% (wt/vol) agarose gels containing 0. 5 μg/ml ethidium bromide and were visualized under UV light.

**Table 1:** PCR primers of human TLRs

Genes	Primer sequence (5'-3')	Amplification size (bp)
TLR1	For: TCTGGTACACGCATGGTC	517 bp
	Rev: ATGGGTGGGAAACTGAAT	
TLR2	For: AACTTACTGGGAAATCCTTAC	264 bp
	Rev: AAAAATCTCCAGCAGTAAAAT	
TLR3	For: GCATTTGTTTTCTCACTCTTT	131 bp
	Rev: TTAGCCACTGAAAAGAAAAAT	
TLR4	For: CGAGGAAGAGAAGACACCAGT	106 bp
	Rev: CATCATCCTCACTGCTTCTGT	
TLR5	For: AGCTTCAACTATATCAGGACA	383 bp
	Rev: TGGTTGGAGGAAAAATCTAT	
TLR6	For: CTTCCATTTTGTTTGCCTTAT	123 bp
	Rev: AGCGGTAGGTCTTTTGGAAC	
TLR7	For: AAACTCCTTGGGGCTAGATG	149 bp
	Rev: AGGGTGAGGTTCGTGGTGTT	
TLR8	For: CTGTGAGTTATGCGCCGAAGA	246 bp
	Rev: TGGTGCTGTACATTGGGGTTG	
TLR9	For: CGCCCTGCACCCGCTGTCTCT	168 bp
	Rev: CGGGGTGCTGCCATGGAGAAG	
TLR10	For: AGAAGAAAGGGAACTGATGAC	279 bp
	Rev: CCTGCCAGTAAATACCAAGT	
GAPDH	For: GGATTTGGTCGTATTGGG	205 bp
	Rev: GGAAGATGGTGATGGGATT	

### Real-time RT-PCR

Real-time RT-PCR was performed to detect TLRs gene expression. The 50 μl reaction mixture contained 45 μl DEPC-H_2_O, 1.0 μl cDNA (1:100 dilution), 2.0 μl (10 μM) of each primer and freeze-dried powder of the AccuPower Greenstar^® ^qPCR premix. The thermal cycle profile for PCR was as follows: 94°C for 5 min, 40 cycles of PCR (94°C for 30 sec; 55°C for 30 sec; 72°C for 30 sec). The fluorescence was digitally collected after each cycle of 72°C for 30 sec. After PCR, the samples were subjected to a temperature ramp with continuous fluorescence monitoring for melting curve analysis. BIONEER Exicycler™ analysis software (Bioneer Corp., Daejeon, Korea) was used to obtain the Ct values. 2^-ΔΔ CT ^method [16] was used to analyze the relative expression of each TLR in MDA-MB-231.

### TLRs protein expression analysis

To detect the cell protein expression of TLRs, 10^6 ^cultured MDA-MB-231 were prefixed and permeabilized. Then, the cells were stained with 3 μl purified anti-human TLR4 antibody (Santa Cruz Biotechnology, CA, USA) at 4°C for 30 min away from light. After washing twice with 1×PBS, the cells were incubated with 2 μl PE-conjugated goat anti-rabbit IgG mAb (Santa Cruz Biotechnology) at 4°C for 30 min away from light, followed by an additional two washes with 1×PBS. Finally, the stained cells in 500 μl 1×PBS were analyzed by using a flow cytometer (FACScalibur; Becton Dickinson (BD), NJ, USA), and the data were processed with BD CellQuest software. The negative control was performed by omitting the anti TLR4 antibody.

### Immunofluorescence analysis

Cells cultured overnight were fixed with alcohol for 30 min and blocked in 1×PBS (pH 7.4) solution with 3% BSA overnight at 4°C in a hydrated box. Anti-TLR4 antibody was added at a 1:100 dilution (Santa Cruz Biotechnology) and allowed to incubate overnight at 4°C in a hydrated box. After washing three times, fluorescent secondary antibody (Santa Cruz Biotechnology) was added at a 1:100 dilution. The cells were again washed three times with 1×PBS, and counter-stained with DAPI. Fluorescence was analyzed by fluorescence microscope (DMI4000B; Leica, IL, USA). Adobe Photoshop 9.0 software (CA, USA) was used for subsequent image processing.

### RNA interference

Cells were transiently transfected with a GFP expressing plasmid pGsil-1 (Genesil, Wuhan, China) containing silencing RNA (siRNA) directed against TLR4. The three pieces of small interfering oligonucleotide specific for human TLR4 have been listed in Table 2 . Briefly, 2×10^5 ^cells were seeded in 6-well dishes and cultured overnight until 60% to 70% confluency was reached. Transfections were performed using Lipofectamine™ 2000 reagent (Invitrogen) per the manufacturer's instructions. Cells were transfected with 4 μg plasmid DNA (TLR4AsiRNA, TLR4BsiRNA, TLR4CsiRNA, vector pGenesil-1 and ScrambledsiRNA) using 8 μl transfection reagent. After 48 h of transfection, fluorescence of cells was observed by a fluorescence microscope. Then, cells were seeded for FCM and immunofluorescence assay. Supernatant was collected to test the inflammatory cytokines secreted by the cells.

**Table 2:** sequences of siRNA against TLR4

Name of siRNA	TLR4 sequences(5'-3')	Site position
TLR4A	a a c t t g t a t t c a a g g t c t g g c	1023-1044
TLR4B	a a g g c t t a c t t t c a c t t c c a a	1374-1395
TLR4C	a a c t c c c t c c a g g t t c t t g a t	1921-1942

### MTT assay

Cells were seeded into 96-well culture plates (6×10^3^/well, 5 wells repeated), allowed to adhere overnight, and then transfections were performed according to the manufacturer's instructions. After 48 h, the transfected cells were collected (0 h) or allowed to continue in culture for 24 h, 48 h, or 72 h. At the end of each treatment, cells were incubated with 5 mg/mL MTT (Sigma Chemical Co., MO, USA) for 4 h and then mixed with dimethyl sulfoxide after the supernatant was removed. The dye absorption (A) was quantitated using an automatic microplate spectrophotometer (340 st; Anthos Zenyth, Salzburg, Austria) at 490 nm.

### Human inflammatory cytokine assay

IL-6 and IL-8 presence in the supernatant of transfected cells were detected according to the instruction of human inflammatory cytokine kit (BD™ Cytometric Bead Array (CBA)). FACScan flow cytometer (BD) was used to analyze samples.

### Statistical Analysis

GraphPad Prism software (CA, USA) was used to perform statistical comparisons between different values. Data were expressed as the means ± standard deviation (SD) with n = 3. Statistical significances were determined by Student's *t-*test and ANOVA, differences were considered significant at a *P *value of less than 0.05.

## Results

### Expression of TLRs in human breast cancer cell line MDA-MB-231

**Figure 1:**
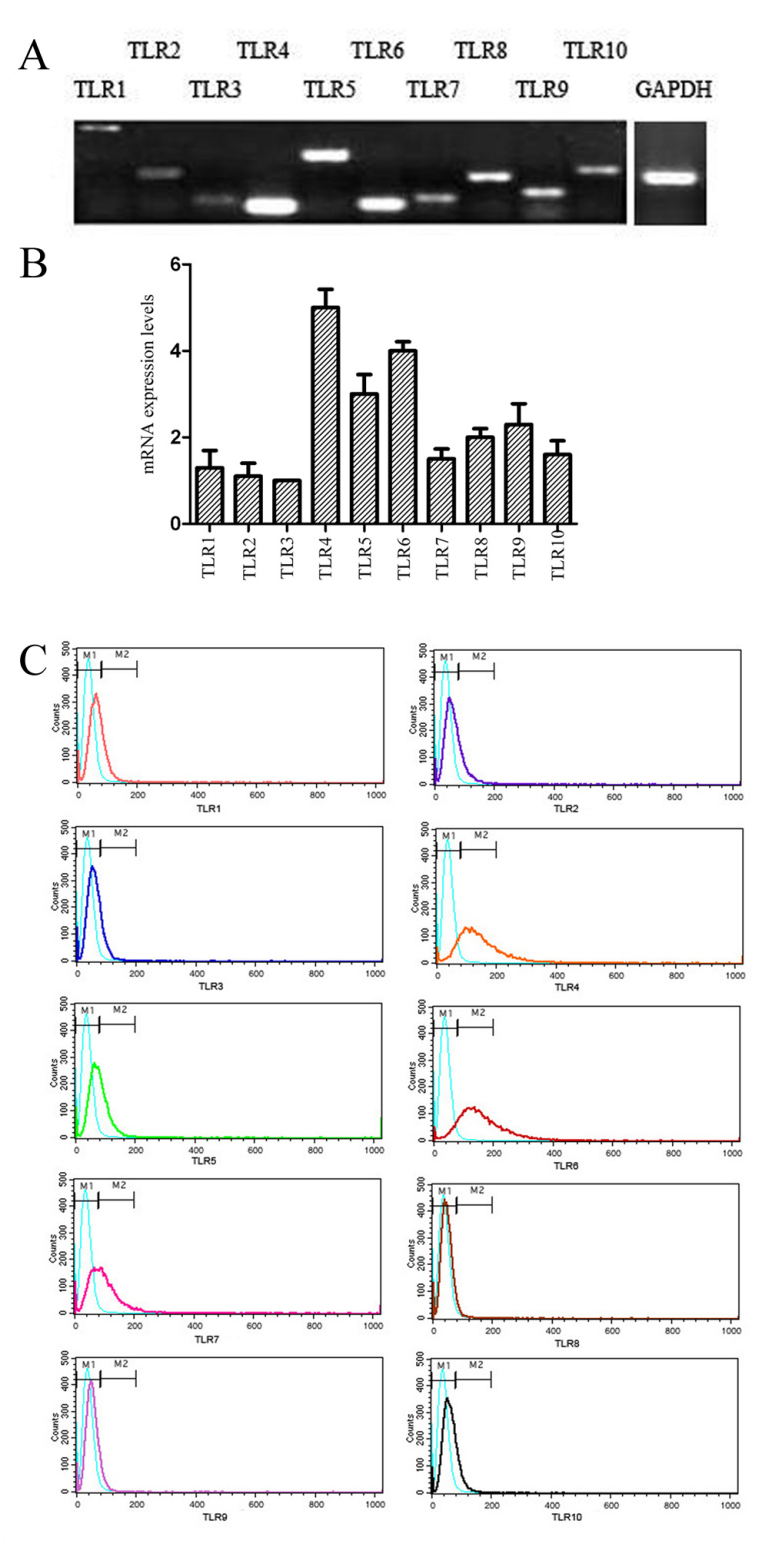
**mRNA and protein expression levels of TLRs (TLR1 through TLR10) in human breast cancer cell line MDA-MB-231**. A, semi-quantitative RT-PCR of TLRs in MDA-MB-231. The GAPDH mRNA was amplified as control. B, real-time RT-PCR of TLRs in MDA-MB-231. The expression of TLR3 was normalized to 1.0 as it was expressed weakest among all TLRs. C, Flow cytometry of TLRs protein expression levels in MDA-MB-231. All results are representative of three separate experiments.

As TLRs have been identified in some tumor cells, we sought to detect if they were expressed in the human breast cancer cell line MDA-MB-231. Qualitative RT-PCR analysis revealed that MDA-MB-231 expressed mRNA of TLR1, TLR2, TLR3, TLR4, TLR5, TLR6, TLR7, TLR8, TLR9 and TLR10 (Figure 1A). Real-time PCR analysis revealed the relative expressions of each TLR examined. The expression of TLR3 was normalized to 1.0, as it was expressed the most weakly. TLR4 was 5-fold higher than TLR3, while other TLRs were expressed between 1- and 4-fold higher than TLR3 (Figure 1B). By FCM detection, we were able to examine the different protein expression levels of the TLRs, TLR4, TLR6, TLR7 and TLR5 were expressed moderately; the other TLRs were expressed weakly or unexpressed. Again, TLR4 protein level was the highest out of TLR1-TLR10 (Figure 1C). Collectively, these results demonstrated that MDA-MB-231 expressed all the TLRs examined (TLR1-TLR10) and TLR4 was expressed highest. TLR4 was strategically selected to investigate its function on the growth and progression of MDA-MB-231 in subsequent studies.

### Efficient knockdown of TLR4 expression by three siRNAs in human breast cancer cell line MDA-MB-231

**Figure 2:**
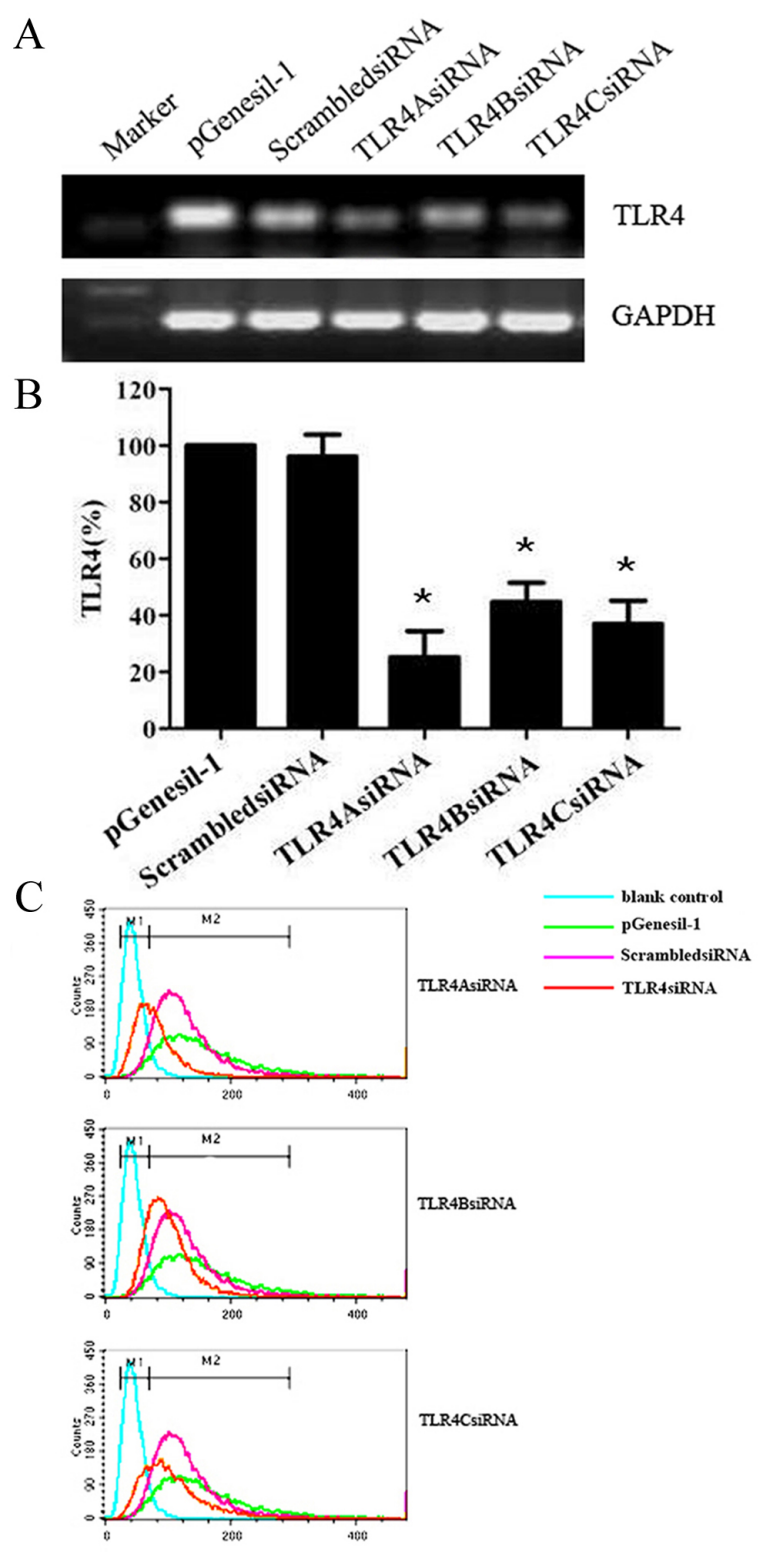
**Transfection and silencing of TLR4 expression using three different siRNAs in human breast cancer cell line MDA-MB-231**. A, RT-PCR of TLR4 from pGenesil-1 vector, ScrambledsiRNA, TLR4AsiRNA, TLR4BsiRNA and TLR4CsiRNA transfected MDA-MB-231. B, the decreased expression of TLR4 at mRNA level in pGenesil-1 vector, ScrambledsiRNA, TLR4AsiRNA, TLR4BsiRNA and TLR4CsiRNA transfected MDA-MB-231 with real-time PCR. C, analysis of transfected cells for TLR4 expression by flow cytometry. All results are representative of three separate experiments.

To study the biological role of TLR4 in the progression of human breast cancer cell line MDA-MB-231, we constructed pGenesil-1 plasmid vectors expressing three different siRNAs directed against TLR4 [GenBank: NM_138554.3] to selectively reduce TLR4 gene expression in MDA-MB-231. The regions have no significant homology to other coactivators or sequences in the human genome database. The vector, TLR4AsiRNA, TLR4BsiRNA, TLR4CsiRNA and ScrambledsiRNA were transfected into MDA-MB-231. After 48 h, the transfected cells appeared to fluoresce green under the fluorescence microscope. Transfection efficiency reached about 70%. From RT-PCR we could see that there were different reductions in TLR4AsiRNA, TLR4BsiRNA, TLR4CsiRNA transfected cells (Figure 2A). Figure 2B showed us that the decreased expression of TLR4 at mRNA levels for TLR4AsiRNA, TLR4BsiRNA and TLR4CsiRNA was 74.8 ± 9.2%, 55.2 ± 6.7% and 63.0 ± 8.3% as compared to vector control (*P *< 0.05). However, no significant difference was observed in siRNA control (*P *> 0.05). As shown in Figure 2C, analysis of the transfected cells for TLR4 expression via FCM demonstrated that specific reductions at protein level for TLR4AsiRNA, TLR4BsiRNA and TLR4CsiRNA was 53.0% ± 2.9%, 37.9% ± 3.7% and 46.7% ± 4.6% as compared to vector control (*P *< 0.05). No obvious difference was seen in siRNA control (*P *> 0.05). Human beast cancer cell line MDA-MB-231 showed that siRNA-directed knockdown of the TLR4 gene was specific. TLR4AsiRNA was the most efficient recombinant plasmid in silencing TLR4 and it was chosen for use in subsequent functional assay.

### TLR4 knock down inhibited proliferation and secretion of inflammatory cytokines in the supernatant of transfected human breast cancer cell line MDA-MB-231

**Figure 3:**
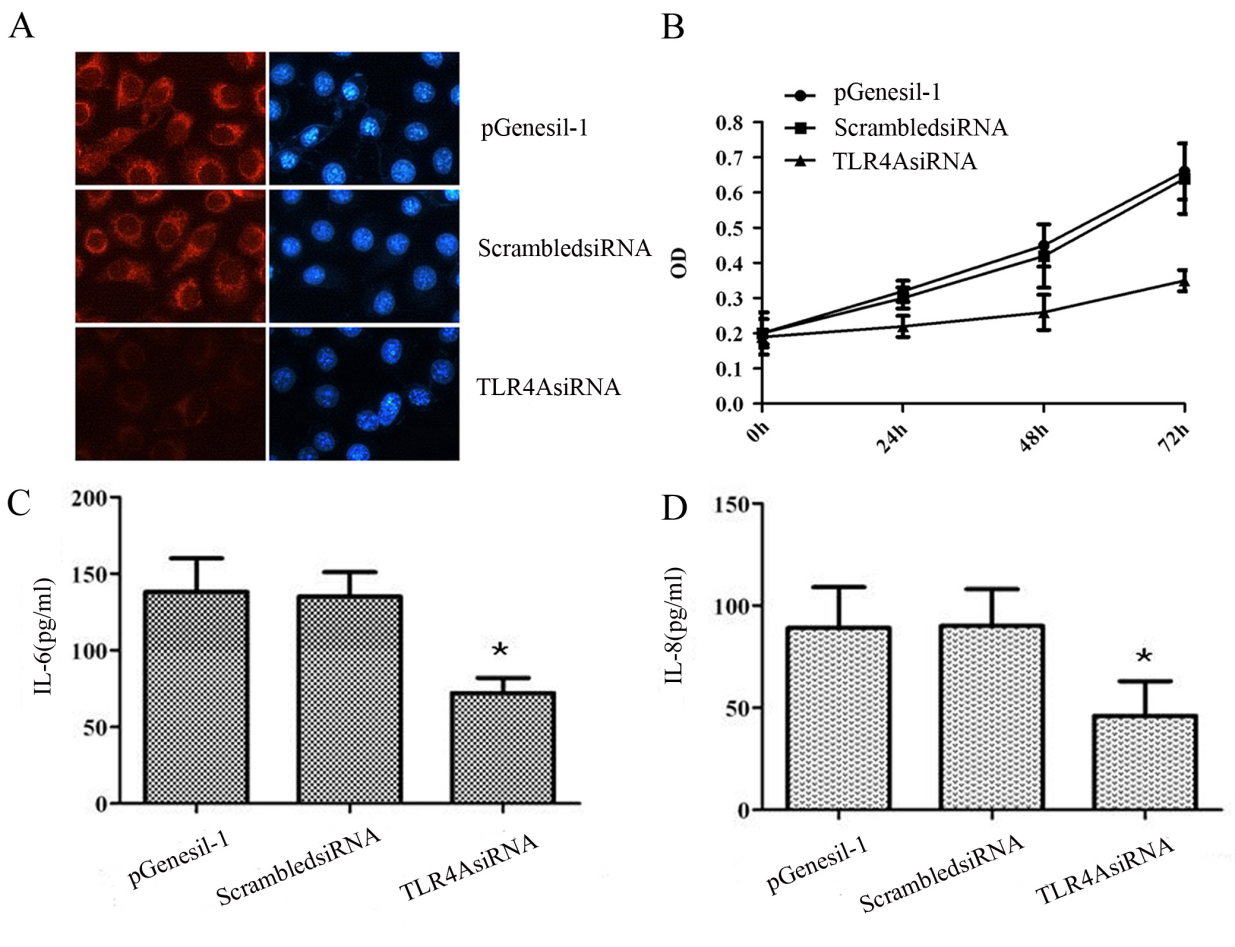
**TLR4 expression and functional effect after TLR4 knockdown in human breast cancer cell line MDA-MB-231**. A, immunofluorescence analysis of gene-specific siRNA on TLR4 protein expression in pGenesil-1 vector, ScrambledsiRNA and TLR4AsiRNA transfected cells. Nuclear staining was performed using DAPI (blue) (200×). B, MTT analysis of the proliferative rate of pGenesil-1 vector, ScrambledsiRNA and TLR4AsiRNA transfected cells. C and D, IL-6 and IL-8 presence in the supernatant secreted by pGenesil-1 vector, ScrambledsiRNA and TLR4AsiRNA transfected cells. Cell supernatant was analyzed using flow cytometry. All results are representative of three separate experiments.

Real-time PCR had demonstrated a specific reduction at mRNA level for TLR4AsiRNA. We further analyzed the effects of TLR4AsiRNA on TLR4 protein expression in MDA-MB-231 using immunostaining with anti-TLR4 antibody. Red fluorescence of TLR4 staining under the fluorescence microscope was drastically reduced by TLR4AsiRNA in comparison to vector control. No obvious difference was seen in siRNA control (Figure 3A). To access the potential effects of TLR4AsiRNA-mediated TLR4 silencing on cell proliferation and survival, MTT analysis was performed on the cells cultured 0 h, 24 h, 48 h, and 72 h following 48 h of transfection. Targeting of TLR4AsiRNA against TLR4 effected the proliferative ability of MDA-MB-231 (Figure 3B). The proliferative rate was significantly decreased according to the time of culture after transfection with TLR4AsiRNA compared with vector control; no significant difference was observed in siRNA control (*P *> 0.05). The biological consequences caused by TLR4 silencing may be a result of changes in TLR4-mediated signaling and subsequent downstream functions. Because increased TLR4 activates TLR4/MyD88 signaling and subsequent downstream functions [17], we decided to examine the status of the TLR4-related inflammatory cytokines in MDA-MB-231 with TLR4 gene knockdown. Analysis of FCM revealed that IL-6 and IL-8 were markedly depressed in the supernatant of silenced cells. The inhibition ration of cytokine IL-6 and IL-8 was 47.8 ± 3.9% and 48.3 ± 4.1% respectively when compared with vector control (*P *< 0.05), no significant difference was seen in siRNA control (Figure 3C and Figure 3D). These results suggested that decreased TLR4 levels in tumor cells might endow cells with attenuated growth and survival capacity.

## Discussion

Recently, much attention has been paid to TLRs and their potential role in different cancers. However, investigations of TLRs and breast cancer are limited. Merrell. *et al*. [10] showed that TLR9 protein is expressed in human breast cancer cells and clinical breast cancer samples. Stimulation of TLR9-expressing breast cancer cells with the TLR9 agonistic CpG oligonucleotides dramatically increased their *in vitro *invasion capacity in both Matrigel assays and three-dimensional collagen cultures. Ilvesaro. *et al*. [18] suggested that TLR9 expression was increased in breast cancer and that CpG oligonucleotide-induced cellular invasion was mediated via TLR9 and TRAF6, independent of MyD88. Xie. *et al*. [19] showed that TLR2 was highly expressed in MDA-MB-231 cells as compared with the MCF-7 breast cancer cell line, and concluded it played a critical role in the cell invasion properties of these cells.

From these studies, we know that TLR9 and TLR2 play a key role in breast cancer proliferation and metastasis. However, the conclusions from different studies are discordant. The growth, proliferation and metastasis of breast cancer are complex and dynamic processes and are likely to be associated with the actions (and interplay) of several TLRs. Not only TLR9 and TLR2, but also other TLRs are involved in the process of breast cancer development. We need to systematically explore the TLR expression profiles of breast cancer cells in order to investigate the relationship between TLRs and the growth, progression and survival of breast cancer cells.

We found that TLRs including TLR1, TLR2, TLR3, TLR4, TLR5, TLR6, TLR7, TLR8, TLR9 and TLR10 were widely expressed in MDA-MB-231 at both the mRNA and protein levels. Real-time PCR analysis and flow cytometry detection showed that TLR4 was the highest expressed. However, the results of TLRs expression of MDA-MB-231 were different from the conclusions of Xie. *et al *[19].

People have reported that TLR4 is an important member of TLRs and has been shown to be present in tumors, such as ovarian cancer [17], prostate cancer cell [20] and colorectal cancer cell [21,22]. The activation of TLR4 expressed on tumor cells may promote tumor growth and resistant of apoptosis. Kelly. *et a1 *[17] found that activation of TLR4 signaling promotes the growth and chemoresistance of epithelial ovarian cancer cells. Blockage of TLR4 signaling has been shown to delay tumor growth and prolong the survival of animals [23,24]. In contrast, in a two-stage chemical carcinogenesis mouse model, in which inflammation mediated the promotion phase of lung cancer, the presence of a functional TLR4 was shown to inhibit lung carcinogenesis, suggesting a protective role of TLR4 in this model of cancer [25]. Therefore, we firstly selected TLR4 to explore whether it was able to either promote or suppress the growth of human breast cancer cell line MDA-MB-231.

Because of the high expression of TLR4 in MDA-MB-231, we choosed RNAi to knockdown the expression of TLR4 to observe the biological character of silenced cells. Three specific pieces of siRNAs successfully decreased TLR4 gene expression and TLR4AsiRNA was the most efficient recombinant plasmid. Functional analysis in our study revealed that the abrogation of TLR4 expression inhibited growth and proliferation strongly. TLR4 played a positive role in the progression of breast cancer cells.

Previous studies have reported that when tumor cells are stimulated with lipopolysaccharides (LPS), the ligand for TLR4, the proinflammatory factors such as nitric oxide, IL-6 and IL-12 are expected to be released from tumor cells, attracting and activating inflammatory cells. Moreover, these factors are known to contribute to the resistance of tumor cells to cytotoxic T lymphocyte (CTL) and natural killer cell (NKC) attack and facilitate evasion from immune surveillance[5]. In our study, TLR4 knockdown *in vitro *lead to TLR4-related inflammatory cytokines being markedly depressed and so it could weaken the ability to the resistance of MDA-MB-231 to CTL and NKC attack and facilitate evasion from immune surveillance. This occurrence *in vitro *may indicate us that TLR4 knockdown *in vivo *could inhibit the growth and promote the death of breast tumors.

## Conclusions

TLR4-mediated cancer growth appears to be an important factor in tumor progression. The use of systemically delivered TLR4-siRNA may provide a novel approach to preventing cancer progression and survival. TLR4AsiRNA directed targeting of TLR4 is a promising candidate for molecular therapy of breast cancer.

## Competing interests

The authors declare that they have no competing interests.

## Authors' contributions

HY participated in study design, carried out most of the experiments, and drafted the manuscript. HQZ participated in its design and coordination. PF participated in FCM analysis. XNZ assisted with cell culture. HYW carried out the molecular genetic studies. XFX carried out the Immunofluorescence analysis. HYS participated in statistical analysis. XMZ conceived of the study, and participated in its design and coordination and helped to draft the manuscript. All authors read and approved the final manuscript.
